# Systematic Review - Combining Neuroprotection With Reperfusion in Acute Ischemic Stroke

**DOI:** 10.3389/fneur.2022.840892

**Published:** 2022-03-17

**Authors:** E. M. Vos, V. J. Geraedts, A. van der Lugt, D. W. J. Dippel, M. J. H. Wermer, J. Hofmeijer, A. C. G. M. van Es, Y. B. W. E. M. Roos, C. M. P. C. D. Peeters-Scholte, I. R. van den Wijngaard

**Affiliations:** ^1^Department of Neurology, The Hague Medical Center, The Hague, Netherlands; ^2^Department of Neurology, Leiden University Medical Center, Leiden, Netherlands; ^3^Department of Radiology and Nuclear Medicine, Erasmus University Medical Center, Rotterdam, Netherlands; ^4^Department of Neurology, Erasmus University Medical Center, Rotterdam, Netherlands; ^5^Department of Neurology, Rijnstate Hospital, Arnhem, Netherlands; ^6^Department of Clinical Neurophysiology, Technical Medical Centre, University of Twente, Enschede, Netherlands; ^7^Department of Radiology, Leiden University Medical Center, Leiden, Netherlands; ^8^Department of Radiology, The Hague Medical Center, The Hague, Netherlands; ^9^Department of Neurology, Amsterdam University Medical Center, Amsterdam, Netherlands

**Keywords:** stroke, neuroprotection, reperfusion after ischemia, thrombectomy, intravenous thrombolysis

## Abstract

**Background:**

Clinical trials of neuroprotection in acute ischemic stroke (AIS) have provided disappointing results. Reperfusion may be a necessary condition for positive effects of neuroprotective treatments. This systematic review provides an overview of efficacy of neuroprotective agents in combination with reperfusion therapy in AIS.

**Methods:**

A literature search was performed on the following databases, namely PubMed, Embase, Web of Science, Cochrane Library, Emcare. All databases were searched up to September 23rd 2021. All randomized controlled trials in which patients were treated with neuroprotective strategies within 12 h of stroke onset in combination with intravenous thrombolysis (IVT), endovascular therapy (EVT), or both were included.

**Results:**

We screened 1,764 titles/abstracts and included 30 full reports of unique studies with a total of 16,160 patients. In 15 studies neuroprotectants were tested for clinical efficacy, where all patients had to receive reperfusion therapies, either IVT and/or EVT. Heterogeneity in reported outcome measures was observed. Treatment was associated with improved clinical outcome for: 1) uric acid in patients treated with EVT and IVT, 2) nerinetide in patients who underwent EVT without IVT, 3) imatinib in stroke patients treated with IVT with or without EVT, 4) remote ischemic perconditioning and IVT, and 5) high-flow normobaric oxygen treatment after EVT, with or without IVT.

**Conclusion:**

Studies specifically testing effects of neuroprotective agents in addition to IVT and/or EVT are scarce. Future neuroprotection studies should report standardized functional outcome measures and combine neuroprotective agents with reperfusion therapies in AIS or aim to include prespecified subgroup analyses for treatment with IVT and/or EVT.

## Introduction

Intravenous thrombolytic therapy (IVT) has become standard care for acute ischemic stroke (AIS), but only a small minority (12%) of patients is eligible for IVT because of the limited time window and contra-indications (1). The absolute benefit of treatment with IVT is limited and is estimated to be 4–10% (2). In the last decade, endovascular therapy (EVT) to mechanically reopen the occluded cerebral artery has led to an improvement of functional outcome in patients with AIS caused by large vessel occlusion (LVO) (3). However, despite high recanalization rates (70–90%) chances of good functional outcome after EVT remain relatively low (30–60%) (3, 4). Currently only 10% of patients after EVT are without stroke symptoms at 3 months follow-up with a modified Rankin Scale (mRS) score of 0 (3, 5). This implies the need for additional treatment and systems-based interventions to further improve recovery of patients with AIS. A wide range of neuroprotective agents has been investigated in the past to reduce brain injury and thereby improve patient recovery. Despite promising results from animal studies, none of the tested neuroprotective strategies appeared effective in clinical trials (6). Earlier trials may have failed due to a lack of recanalization in treating patients with AIS. As ischemic tissue will eventually become infarcted if blood flow is not restored, adequate reperfusion is probably a necessary condition for recovery with or without additional neuroprotective treatments (4, 7–9). The four primary treatment targets are reduction of excitotoxicity, oxidative stress, inflammation, and cellular apoptosis (10). In patients with adequate recanalization, another targeted mechanism is reducing reperfusion injury (7, 11). With the introduction of IVT and EVT, drugs with neuroprotective properties can now be investigated in combination with reperfusion therapy. This systematic review provides an overview of randomized controlled trials (RCTs) of neuroprotective agents in AIS as an adjunct to IVT and/or EVT.

## Methods

### Search Strategy

This systematic review adhered to The Preferred Reporting Items for Systematic reviews and Meta-Analyses (PRISMA) 2020 statement and focused on RCTs in patients with AIS undergoing reperfusion therapy and neuroprotective therapy within 12 h of stroke onset. We selected studies by searching electronic databases (PubMed, Embase, Web of Science, Cochrane Library, Emcare) up to and including September 23rd 2021 using the search query specified in Box 1 (Supplementary Material). Two authors (EMV and VJG) independently screened the search results by reading titles and abstracts. All disagreements between raters were included for full text screening. Full text screening was done by two authors authors (EMV and IRW), all disagreements were re-evaluated until consensus was achieved.

### Selection Criteria

**Figure 1:**
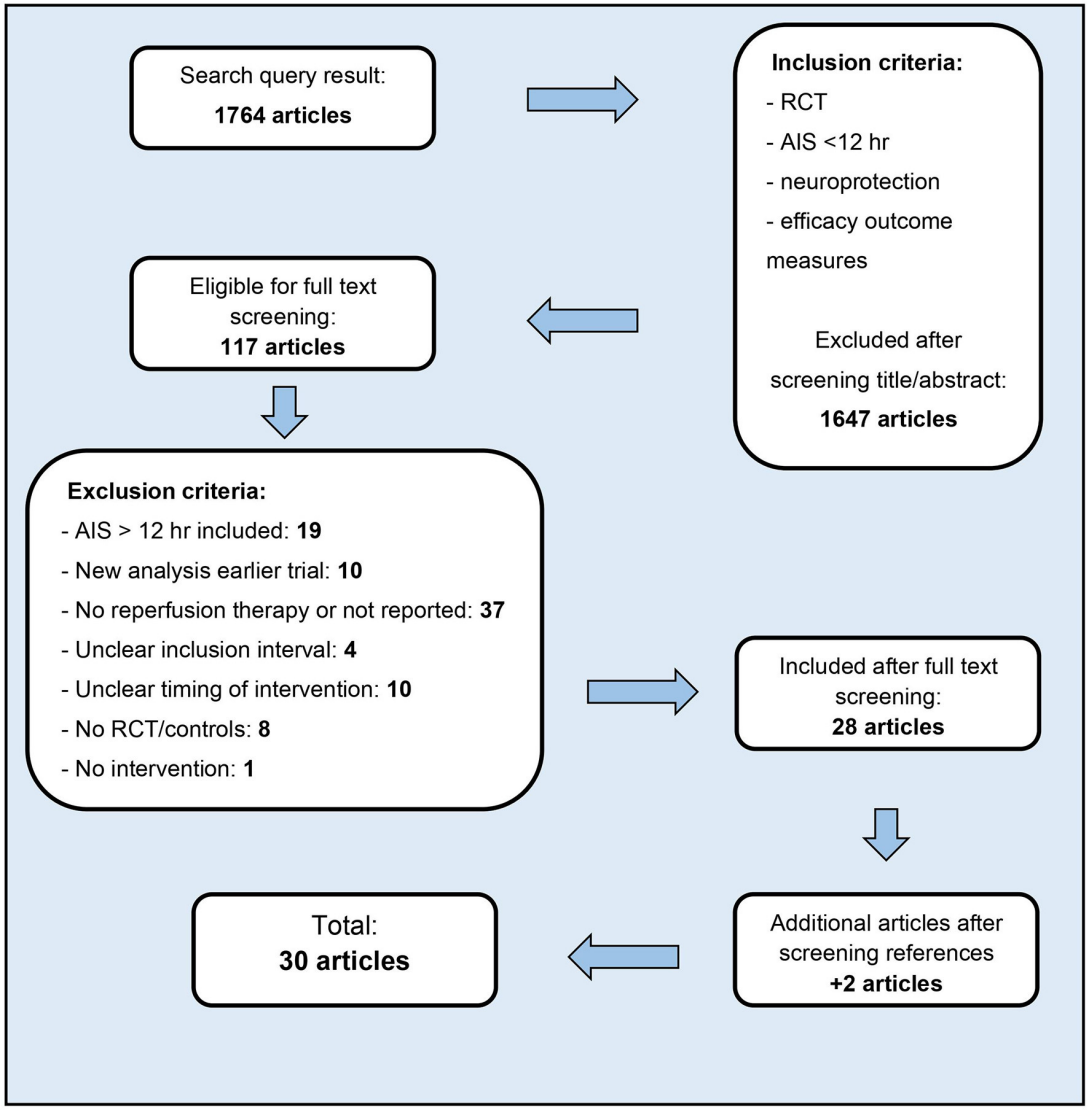
Article selection.

RCTs studying neuroprotective agents in patients with AIS in addition to any form of reperfusion therapy within 12 h of stroke onset were selected. Exclusion criteria included non-English articles, an unclear inclusion interval or unclear timing of treatment with the neuroprotective agent, and articles not reporting exact numbers of patients treated with reperfusion treatments (Figure 1). The reference lists of all relevant articles were screened for additional studies.

### Data Extraction

Analyzed primary outcomes were mRS, National Institutes of Health Stroke Scale (NIHSS), Barthel Index (BI), mortality, and risk of hemorrhage. Analyzed secondary outcomes were radiological markers including rate of recanalization measured with the thrombolysis in cerebral infarction (TICI) grading scale, and infarct volume measured by CT or MRI if available. Odds ratios (OR) for good functional outcome (mRS 0–2 vs. 3–6) and mortality (mRS 6) were calculated with reported mRS score distributions if available. All populations were assessed for type of reperfusion therapy (IVT, EVT, or both) and percentage of LVO. When no data were available on percentage of LVO in the cohort, associated radiological information was documented. Time to reperfusion therapy was noted. Neuroprotective treatment regimens were extracted including time between start of reperfusion therapy and neuroprotective treatment. Whether neuroprotective treatment was started before or after reperfusion therapy was noted. Results were grouped according to the main mechanism of action.

### Availability

The extraction form is available in the Supplementary Material. All data may be shared upon request.

### Assessment of Bias

All included articles were evaluated with the modified Cochrane collaboration tool to assess risk of bias for RCTs.

### Role of the Funding Source

The authors received no funding for this study. Funders of the trials included in this review did not have any influence on the design, interpretation, and reporting of this review.

## Results

**Figure 2:**
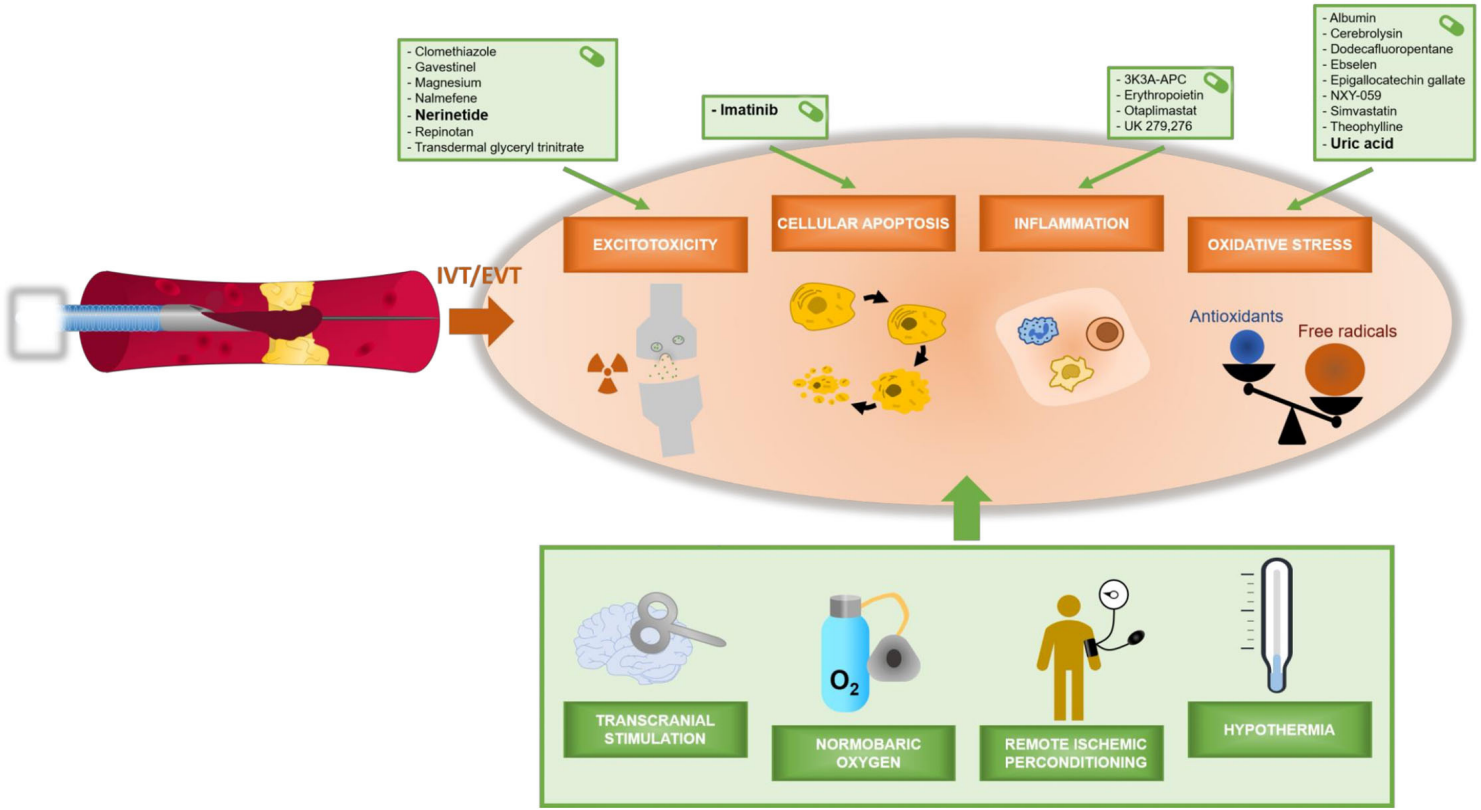
Overview of neuroprotective agents and their assumed mechanisms of action.

The search performed on September 23rd 2021 (initial search date: November 13th 2020) yielded 1,764 articles, of which 117 were selected for full text screening. Ultimately, 30 studies were included in this systematic review, encompassing a total of 16160 patients. Results are presented in Tables 1, 2, subdivided into studies combining neuroprotection with either IVT or EVT, and further categorized by presumed mechanism of action: reduction of excitotoxicity, oxidative stress, inflammation, cellular apoptosis, and other/non-pharmacological. Most neuroprotective agents have an effect on multiple mechanisms. Figure 2 shows an overview of neuroprotective agents and their assumed mechanisms of action. In the majority of studies (26/30), patients were treated within minutes to several hours after IVT or before completion of EVT. Three studies reported on pre-hospital administration of neuroprotective treatment; only one specifically administered neuroprotective treatment after recanalization with EVT. Fifteen studies were specifically designed to investigate effects of neuroprotective treatment in addition to reperfusion therapy, i.e., all patients had to receive reperfusion therapy, either IVT and/or EVT. Of these 15 studies with 100% reperfusion therapy, in only 3 studies all patients were treated with EVT. Five of these 15 studies showed a positive result of neuroprotective treatment on functional outcome. In the other 15 studies not all patients were treated with reperfusion therapy. The number of patients treated with IVT or EVT was limited or predefined subgroup analyses on patients receiving IVT or EVT were not reported. Our calculated ORs for good clinical outcome (mRS 0–2) did not reach statistical significance in any of the reported trials. The OR for mortality (mRS 6) was only significantly lower in the study reporting on normobaric oxygen. An overview of unadjusted ORs for good functional outcome and mortality is presented in the Supplementary Material. Risk of bias assessment is presented in Table 3. Studies in which <25% of patients was treated with IVT are summarized in Tables 1, 2, but not separately mentioned in the results below.

**Table 1:** Overview of neuroprotective trials using reperfusion therapy.

**Study**	**Year**	**Agent**	**Mechanism of action**	**Reperfusion therapy**	**Patients (controls)**	**Mean age (control)**	**Female % (control)**	**Median baseline NIHSS (controls)**	**% LVO/TACS (controls)**	**Median time to treatment in hr (controls)**	**Median time to IVT/EVT in hr (controls)**
Ogawa et al. (12)	1999	Ebselen	Antioxidant, anti-inflammatory	IVT (73%)	99 (56)	66.6 (65.6)	33% (32%)	unknown	MCA 100%	8.8 (8.4)	IVT 4.6 (6.2)
Clark et al. (13)	2000	Nalmefene	K-receptor opiate antagonist	IVT (9%)	368 (182)	69.8 (70.5)	49.7% (48.5%)	12.2 (12.4)	No LVO reported	4.8 (4.2)	unknown
Sacco et al. (14)	2001	Gavestinel	Glycine antagonist	IVT (24%)	1376 (666)	70 (70)	47.8% (47.4%)	12 (12)	TACS 37.7%	5.2 (5.2)	unknown
Lyden et al. (15)	2001	Clomethiazole	GABA enhancer	IVT (100%)	190 (93)	68 (67.6)	44% (46%)	14 (13)	TACS 53% (41%)	5.4 (5.3)	unknown, all but 2 IVT <3 h
Krams et al. (16)	2003	UK 279,276	Anti-inflammatory	IVT (21%)	966 (248)	72 (total)	43% (total)	13 (unknown)	No LVO reported	3.1 (unknown)	IVT 2.3 (unknown)
Amaro et al. (17)	2007	Uric acid	Antioxidant	IVT (100%)	24 (8)	70.2 (70.2, 72.9)	0% (38%, 50%)	11.8 (8.5)	No LVO reported	3.6 (3.5)	IVT 2.3 2.3 (2.1)
Diener et al. (18)	2008	NXY-059	Antioxidant	IVT (39%)	4946 (2478)	71 (71)^**^	44.4% (45.8%)	12 (12)	No LVO reported	3.45 (3.45)	unknown
Ehrenreich et al. (19)	2009	Erythropoietin	Antioxidant, anti-inflammatory	IVT (63%)	522 (266)	68.6 (68.2)	45% (47%)	13 (12)	LVO 22.7% (25.4%)	4.4 (4.5)	unknown
Teal et al. (20)	2009	Repinotan	Serotonin receptor agonist	IVT (61%)	681 (337)	70.3 (69.7)	48% (47%)	15 (14)	No LVO reported	unknown	unknown
Hemmen et al. (21)	2010	Hypothermia	Hypothermia	IVT (79%)	58 (30)	68.9 (62.3)	43.3% (46.2%)	14.3 (13.7)^*^	No LVO reported	1.1 (N/A)	unknown
Heiss et al. (22)	2012	Cerebrolysin	Antioxidant, anti-inflammatory	IVT (9%)	1067 (540)	65 (65.5)	40.4% (39.6%)	9 (9)	No LVO reported	7.7 (7.6)^*^	unknown
Ginsberg et al. (23)	2013	Albumin	Antioxidant, anti-inflammatory, hemodilution	IVT (68%) EVT (4%) IVT + EVT (16%)	841 (419)	63.4 (64.8)	48% (44%)	11 (11)	TACS 24% (23%)	3.3 (3.3)	IVT 2.1 (2.2)
Lang et al. (24)	2013	Cerebrolysin	Antioxidant, anti-inflammatory	IVT (100%)	119 (59)	65.5 (67)	33.3% (37.3%)	12.3 (11.0)*	No LVO reported	unknown, all 1 h after IVT	IVT 2.4 (2.2)^*^
Hougaard et al. (25)	2014	Remote ischemic perconditioning	Antioxidant, anti-inflammatory	IVT (100%) IVT + EVT (unknown)	443 (196)	66 (68)^**^	43% (41%)	4 (5)	No LVO reported	unknown, pre-hospital	unknown
Saver et al. (26)	2015	Magnesium	Calcium-channel blocker, NMDA antagonist, vasodilatation	IVT (36%)	1700 (843)	69 (69)	43.5% (41.8%)	11.5 (11.2)	No LVO reported	0.75 (0.75)	unknown
Woodhouse et al. (27)	2015	Transdermal Glyceryl Trinitrate	Nitric oxide donor	IVT (34%)	273 (139)	69 (70.8)	47.9% (38.8%)	11.4 (12.4)	TACS 29.9% (33.3%) LVO	unknown	unknown
Montaner et al. (28)	2016	Simvastatin	Antioxidant, anti-inflammatory	IVT (53%)	104 (54)	73.5 (75)^**^	52% (40.7%)	7 (7)	TACS 22% (22.2%)	7.4	unknown
Wahlgren et al. (29)	2017	Imatinib	Tyrosine kinase inhibitor	IVT (100%) EVT (42%)	60 (17)	76/73/72 (70)^**^^†^	57%/27%/47% (35%)^†^	12 (13)	No LVO reported	4.0 (unknown)	IVT 1.4 (all patients) EVT unknown
Wang et al. (30)	2017	Epigallocatechin gallate	Antioxidant	IVT (100%)	371 (186)	62.7 (65.1)/67.4 (64.9)^†^	49% (46%)/45% (49%)^†^	13 (15)	No LVO reported	unknown	unknown
Chamorro et al. (31)	2017	Uric acid	Antioxidant	IVT + EVT (100%)	45 (21)	78 (68)^**^	46% (33%)	17 (15)	IC/T 17% (25%) M1 67% (55%) M2 13% (20%)	2.3 (2.5)	IVT 1.9 (1.8)
Lyden et al. (32)	2019	3K3A-APC	Anti-inflammatory, anti-thrombotic	IVT 54% EVT 5% IVT + EVT 42%	110 (44)	64 (64)	56% (45%)	13 (13.5)	No LVO reported	unknown	2.2 (2.1)
Culp et al. (33)	2019	Dodecafluor- opentane	Improved oxygen transport	IVT 58% EVT 4% IVT + EVT 25%	24 (6)	56.9 (55.8)	39% (17%)	6.5 (9.5)	No LVO reported	6.3 (6.6)	unknown
Kim et al. (34)	2020	Otaplimastat	Anti-inflammatory	IVT 100% EVT 30%	69 (24)	63.5/66 (59)^**^^†^	43%/23% (32%)^†^	8 (10)	No LVO reported	1.8 (2.0)	1.5 (1.8)
Pico et al. (35)	2020	Remote ischemic perconditioning	Antioxidant, anti-inflammatory	IVT 57% EVT 4% IVT + EVT 30%	188 (95)	67.8 (66.7)	51.6% (44.2%)	9 (10)	MCA 48.4% (51.6%) Cervical carotid 6.5% (0%) Carotid tandem 6.5% (5.3%) Other 6.5% (12.6%)	3.7 (N/A)	IVT 2.6 (2.4) EVT 3.1 (3.0)
Hill et al. (36)	2020	Nerinetide	Reduction of intracellular endogenous nitric oxide	IVT 60% EVT 100%	1105 (556)	71.5 (70.3)^**^	48.8% (50.5%)	17 (17)	ICA 20% (18.5%) MCA 80.7%	3.1 (3.1)	unknown
An et al. (37)	2020	Remote ischemic perconditioning	Antioxidant, anti-inflammatory	IVT 100%	68 (34)	62.1 (67.1)	35.3% (26.5%)	6.5 (4.5)	No LVO reported	3.0 (3.0)	unknown
Modrau et al. (38)	2020	Theophylline	Redistribution of blood	IVT 100% EVT 14%	64 (31)	68 (71)^**^	39% (39%)	8 (6)	LVO 42% (39%) ACA 0% (3% M1 22% (17%) M2 19% (17%) PCA 3% (3%)	unknown	unknown
He et al. (39)	2020	Remote ischemic perconditioning	Antioxidant, anti-inflammatory	IVT 100%	49 (25)	59.5 (61.3)	16.7% (28%)	7 (9)	No LVO reported	3.0 (3.2)	unknown
Cheng et al. (40)	2021	Normobaric oxygen	Antioxidant	IVT 45% EVT 100%	175 (87)	63.8 (65.9)	36.4% (41.4%)	17 (16)	MCA 56.8% (66.7%) ICA 21.6% (17.2%)	unknown	unknown
Pruvost-Robieux et al. (41)	2021	Transcranial direct current stimulation	modulates excitability of neurons	IVT 56% EVT 7% IVT + EVT 38%	55 (23)	71.6 (76.2)	36.4% (56.5%)	8 (11)	Proximal (ICA, M1, M2) 37% (57%) Distal 18% (13%) None 45% (30%) Prox. ICA 21.6% (16.1%)	2.8 (2.9)	IVT 2.2. (2.3) EVT 2.6 (2.8)

* *mean, not median*;

** *median, not mean*

† *multiple treatment groups*.

*IVT, intravenous thrombolysis; EVT, endovascular therapy; LVO, large vessel occlusion; NIHSS, National Institutes of Health Stroke Scale; TACS, total anterior circulation syndrome. IC/T, Internal carotid/tandem occlusion; M1, proximal MCA M1 part; M2, proximal MCA M2 part*.

**Table 2:** Interventions, outcome measures, and results in neuroprotective trials.

**Agent**	**Method and duration of administration**	**Primary outcome measures**	**Secondary outcome measures**	**Results**	**Significant safety issues?**
Ebselen	Oral administration <12 h of stroke onset, continued for 14 days	- CT infarct volume at 30 days - GOS at 30 days	- Modified Mathew scale at 30 days	No significant differences in primary or secondary outcomes in ITT analysis	No
Nalmefene	60 mg IV bolus dose of nalmefene followed by 50 mg continuous infusion for 24 h, started <12 h of stroke	- Proportion of patients with BI ≥ 60 at 3 months and a rating “moderate disability” or better on the GOS at 3 months	- NIHSS at 3 months - NIHSS success rate (≥4 points decrease from baseline) at 3 months - Success rates for BI at 3 months, GOS at 3 months, and combined BI+GOS at 3 months by patient age (< or ≥ 70 years)	Primary outcome did not differ for nalmefene treatment (66.9%) vs. placebo (62.3%) (*p* = 0.46). No treatment effect after 3 months on any of the outcome measures	No
Gavestinel	1,800 mg IV administered over three days started <6 h of stroke onset	- BI at 3 months, trichotomized to “independent = 95–100”, “assisted independence = 60–90” and “dependence = 0–55”	- BI at 7 days and 1 month - NIHSS at 1 and 3 months - mRS at 1 and 3 months	No improvement on BI trichotomy (*p* = 0.79), BI score 95–100 was 39% in gavestinel group vs. 37% in placebo. No difference in 3-month survival (Kaplan-Meier, *p* = 0.11). No other secondary outcome suggested benefit from gavestinel.	No
Clomethiazole	68 mg/kg IV in 24 h started <12 h of stroke	- Deaths - Adverse and serious adverse events	- BI - NIHSS - SSS - mRS	SAE 48 in treated group vs. 47 in placebo group, no significant differences in functional outcome	No
UK 279,276	Single IV infusion over 15 min <6 h of stroke onset	- SSS change baseline to 90 days	- NIHSS change baseline to 90 days - BI at 90 days - mRS at 90 days	No improved outcome on any of the efficacy parameters (posterior probability of futility, 0.89). The trial was stopped early for futility.	No
Uric acid	500 or 1,000 mg IV over 90 min started <3 h of stroke	- Safety at 90 days, primary safety end point was the rate of gout attack	- Mean NIHSS at 90 days - mRS at 90 days - vascular death at 90 days	No SAE in uric acid treated group. No significant difference in NIHSS (*p* = 0.24), mRS (*p* = 0.26) or vascular death (*p* = 0.58)	No
NXY-059	IV infusion over 72 h started <6 h of stroke onset	- Distribution of disability scores on the mRS at 90 days	- Neurologic and activities of daily living scores - Risk of Alteplase related hemorrhage	The distribution of scores was not different in the group treated with NXY-059 compared with placebo (OR for limiting disability 1.02; 95% CI, 0.92–1.13, *P* = 0.682). No decrease in rates of hemorrhage in IVT treated patients.	No
Erythropoietin	40.000IU IV at 0, 24, and 48 h after stroke, started <6 h of stroke onset	- BI at 90 days	- mRS at day 90 - NIHSS at day 90 - MRI variables	No effect om primary outcome (ITT: EPO vs. placebo: 56.9 ± 42 vs. 59.2 ± 41; 95% CI,−4.41 to 9.86; *P* = 0.45). Subgroup analysis shows improved delta NIHSS (day 1 minus day 90) in EPO group in the PP non-rtPA group vs. placebo (mean difference of 5.3 ± 5.3 in EPO vs. 3.2 ± 6.4 placebo; *P* <0.03)	More deaths in the EPO group (16.4 vs. 9.0%; OR, 1.98; CI, 1.16–3.38; *P* <0.01)
Repinotan	Continuous 72-h intravenous infusion started within 4.5 h of stroke onset	- BI ≥85 at 3 months	- mRS 0,1, or 2 at 3 months - NIHSS decrease ≥4 points at 3 months	BI ≥85 in 37.1% (repinotan) vs. 42.4% (placebo), (CMH probability value = 0.149). mRS 32.2 vs. 37.1% (CMH = 0.169). NIHSS decrease ≥4 66.7 vs. 69.6% (CMH = 0.413)	No
Hypothermia	24 h of endovascular cooling to 33°C followed by 12 h of controlled rewarming/normothermia started <6 h of stroke onset	- SAE at 3 months - Achievement of cooling to 33°C	- Incidence of hemorrhage at 36 h - Mortality at 90 days - NIHSS at 1, 30, and 90 days - mRS 0 or 1 at 90 days	Cooling was achieved in all but 2 patients due to technical difficulties. mRS 0 or 1 at 3 months 18% (hypothermia) vs. 24% (normothermia) (*p* <0.77). NIHSS mean at 90 days: 6.3 vs. 3.8 (*p* = 0.355)	Pneumonia more frequent after hypothermia (50 vs. 10%, *p* = 0.001)
Cerebrolysin	Intravenous infusion for 10 days started <12 h of stroke onset	- BI, mRS, and NIHSS evaluated in 1 global test at 90 days	- Responder analysis based on responder definitions for mRS, BI, and NIHSS.	Median NIHSS improvement of 6 (vs. 5 placebo), median BI improvement of 30 points (vs. 30 points), and final mRS 2 (vs. 2). No significant differences.	No
Albumin	2 g/kg IV administration over 2 h, started within 90 min of IV Alteplase	- Favorable outcome at 90 days defined as mRS 0 or 1, NIHSS 0 or 1, or both	- mRS - NIHSS - BI - Stroke specific QOL scale - Trailmaking A and B test	Favorable outcome at 90 days 44% (albumin) vs. 44% (placebo). RR 0.96 (CI 0.84–1.10). No significant differences in any of the secondary outcome measures	More pulmonary oedema and congestive heart failure in albumin treated group (13 vs. 1%, RR10.8 CI 4.37–26.72)
Cerebrolysin	IV administration in 30 min, continued for 10 consecutive days, started directly after IVT	- mRS at 90 days - Responder analysis (mRS 0 or 1 vs. 2–6)	- NIHSS change - BI - GOS	No significant differences in mRS (*p* = 0.629), NIHSS change (*p* = 0.981), BI (*p* = 0.988) or GOS (*p* = 0.845) at 90 days including responder analyses	No
Remote ischemic perconditioning	4 cycles of inflation/deflation during ambulance transport	- Penumbral salvage, defined as the volume of the perfusion–diffusion mismatch not progressing to infarction after 1 month.	- Final infarct size - Infarct growth - Clinical outcome at 3 months	Penumbral salvage (11.89 vs. 14.10 ml *p* = 0.20), final infarct size at 1 month (1.63 vs. 1.99 ml *p* = 0.97), infarct growth between baseline and 1 month (0 vs. 0.02 ml *p* = 0.79), and clinical outcome after 3 months did not differ among groups.	No
Magnesium	IV loading dose of magnesium sulfate followed by 24 h continuous infusion, started within 2 h of stroke onset	- mRS at 90 days	- Excellent outcome defined as mRS 0 or 1, NIHSS 0 or 1, BI ≥ 95, or GOS 1 at 90 days - Good outcome defined as modified Rankin score ≤ 2, Barthel Index ≥60, NIHSS score ≤ 8, and GOS score of 1 or 2 - Neurological deficit at 90 days (NIHSS)	No significant shift in mRS between magnesium treated patients and placebo (*p* = 0.28), no difference in mean mRS at 90 days (2.7 vs. 2.7, *p* = 1.00). No benefit of magnesium in prespecified secondary end points.	No
Transdermal Glyceryl Trinitrate	Single dose of transdermal glyceryl trinitrate within 6 h of stroke onset	- mRS at 90 days	- Intracerebral hemorrhage at 7 days - Recurrent stroke - NIHSS - SSS	mean mRS score 2.6 vs. 3.2, mean difference 0.65 (CI 0.22–1.08 *p* = 0.003). No significant treatment effect of glyceryl trinitrate in specified subgroups including use of Alteplase.	No
Simvastatin	Oral simvastatin 40 mg once daily for 90 days, started <12 h of stroke onset	- Proportion of independence defined as mRS ≤ 2 at 90 days	- NIHSS reduction at 90 days - Proportion of patients with NIHSS reduction ≤ 4 points at 7 days	Proportion of patients with mRS ≤ 2 at 90 days: 68.8% (simvastatin) vs. 70% (placebo), OR 0.99 (CI 0.35–2.76, *p* = 0.98). No significant differences in secondary outcomes.	No
Imatinib	Oral imatinib in three dose levels (400;600;800 mg) for 6 days	- Adverse events	- Hemorrhagic transformation - Infarct volume - Cerebral oedema - NIHSS at day 7 - mRS 0–2 at 90 days	- NIHSS improvement per dose group compared with controls, adjusted for EVT (low, median, high): 2 (*p* = 0.259), 3 (*p* = 0.106), 5 (*p* = 0.012) - mRS 0–2 72% (all treatment groups) vs. 61% controls (*p* = 0.296)	No
Epigallocatechin gallate	500 mg of epigallocatechin administered simultaneously with IVT, as a 10% bolus followed by continuous infusion of 1 h daily, for a total of 7 days.	- NIHSS at 1 day - NIHSS at 7 days	- Plasma levels MMP-2 and MMP-9 at day 1 and 7	No significant differences in early treated patients (0–3 h), NIHSS shift in favor of epigallocatechin in patients treated 3–5 h after onset, though no p-values reported.	No
Uric acid	1,000 mg intravenous infusion	- Rate of good outcome defined as mRS 0–2 at 90 days	- Shift analysis mRS - BI ≥ 95 at 90 days - NIHSS increment ≥ 4 points - Infarct growth - Infarct volume	mRS 0–2, 67% (uric acid) vs. 48% (placebo) (adjusted OR 6.12 95% CI 1.08−34.56). No significant shift in mRS. BI ≥ 95 67 vs. 43% (adjusted OR 9.20 95% CI 1.53 – 55.20). Infarct volume was nonsignificantly smaller in uric acid group, but infarct growth was reduced.	No
3K3A-APC	Intravenous administration of 3K3A-APC in different dose tiers up to 540 ug/kg, started after IVT	- Dose limiting toxicities	- Hemorrhage at 30 days - Microbleed incidence at 30 days - mRS 0 or 1 at 90 days - BI ≥ 90 at 90 days	- No difference in prespecified dose limiting toxicities (*p* = 0.43) - No difference in 90-day mRS, including all dose tiers together (45.2% treatment vs. 62.8% placebo). - No difference in BI ≥ 90 at 90 days 76.9 vs. 91.9%. ]-No difference in hemorrhage (*p* = 0.54) or microbleeds in infarct zone (*p* = 0.28)	No
Dodecafluoropentane	3 intravenous doses, 1 every 90 min, started <12 h of stroke onset	- SAE - NIHSS - mRS		mRS at 30 and 90 days significantly decreased comparing high-dose group vs. placebo (*p* = 0.01 and *p* = 0.03), though at baseline less severe strokes in the cohort of high-dosed patients.	No
Otaplimastat	40 or 80 mg administered intravenously twice daily, for a duration of 3 days, started <30 min after IVT	- Incidence of parenchymal hematoma <24 h	- Symptomatic intracranial hemorrhage (NIHHS detoriation ≥ 2 points) <5 days - Incidence of major systemic bleeding - mRS at 90 days - BI at 90 days - NIHSS change at 90 days - Infarct growth	- No difference in primary outcome or other bleeding complications - mRS at 90 days placebo vs. otaplimastat 40 mg: median 1.0 vs. 0.0, adjusted OR 3.2 (CI 0.9 – 10.9, *p* = 0.068). - mRS 90 days placebo vs. otaplimastat 80 mg 1.0 vs. 1.0, adjusted OR 2.0 (CI 0.6 – 6.7, *p* = 0.246) - No significant change in NIHSS (p>0.999)	No
Remote ischemic perconditioning	4 cycles of cuff inflation to 110 mmHg above systolic blood pressure for 5 min started <6 h of stroke onset	- Change in brain infarction volume at 24 h measured by DWI	- Relative change infarct volume (%) - NIHSS change at 24 h - BI ≥ 95 at 90 days - mRS 0 or 1 at 90 days - Successful recanalization defined as TICI scale 2b or 3	- No significant difference in primary or secondary outcomes - Median absolute infarct change 0.30 cm^3^ in treatment group vs. 0.37 cm^3^ controls (mean log−0.07, CI−0.33–0.18). - Excellent outcome at 90 days 51.1 vs. 40.7% (*p* = 0.12)	No
Nerinetide	Single intravenous dose 2.6 mg/kg administered in 10 min	- Good outcome defined as mRS 0–2 at 90 days	- BI ≥ 95 at 90 days - NIHSS 0–2 at 90 days - Excellent outcome defined as mRS 0 or 1 - Mortality rates	- Subgroup of patients not treated with Alteplase with better outcome, suspected interaction between nerinetide and Alteplase nullifying treatment effect. - mRS 0–2: adjusted RR 1.04 (CI 0.96–1.14) - NIHSS 0–2: adjusted RR 1.03 (CI 0.92–1.11) - mortality: adjusted RR 0.84 (CI 0.63–1.13) - mRS 0–2 in no Alteplase stratum: adjusted RR 1.18 (CI 1.01–1.38)	No
Remote ischemic perconditioning	5 cycles of inflation to 180 mmHg for 5 min on both arms, twice daily continued during hospital stay, started <3 h of IVT	- Favorable outcome defined as mRS 0 or 1 at 90 days	- mRS 0–2 at 90 days - NIHSS 0–2 at 90 days - BI ≥ 95 at 90 days	- mRS 0 or 1 at 90 days: 71.9 vs. 50% controls, adjusted OR 9.85 (CI 1.54–63.16, *p* = 0.016) - mRS 0–2 at 90 days: 81.3 vs. 58.8%, adjusted OR 8.25 (CI 1.4–48.7, *p* = 0.02) - NIHSS improvement ≥6 at 90 days: 41.9 vs. 16.1%, adjusted OR 8.2 (CI 1.17–17.75, *p* = 0.03)	No
Theophylline	220 mg theophylline intravenously administered in 15 min, started <30 min of IVT	- Early clinical improvement as absolute improvement in NIHSS score at 24 h - Infarct growth at 24 h at MRI	- Major clinical improvement at 24 h defined as NIHSS reduction ≥50% - mRS 0 or 1 at 90 days	- NIHSS improvement at 24 h: 4.7 (SD 5.6) in theophylline patients, vs. 1.3 (SD 7.5) placebo (*p* = 0.044), adjusted OR 3.6 (CI 0.1–7.1, *p* = 0.043) - Infarct growth 141.6% in theophylline group vs. 104.1% (*p* = 0.146) - NIHSS improvement >50% in 67 vs. 45% controls (*p* = 0.07) - mRS 0 or 1 at 90 days: 61 vs. 58% (*p* = 0.802)	No
Remote ischemic perconditioning	4 cycles of inflation to 200 mmHg for 5 min on the healthy arm at 6 and 18 h after IVT	- Rate of hemorrhagic transformation <7 days - Adverse events - hs-CRP and hcy at 24 h - Blood pressure in the first 24 h	- Distribution of mRS at 90 days - Ratio of mRS 0–1 at 90 days - NIHSS at 24 h, discharge/7 days and 30 days	- mRS ≤ 1 at 90 days 62.5 vs. 68% controls (*p* = 0.686) - No significant difference in mRS at 90 days (*p* = 0.350) - No significant difference in NIHSS at 24 h, 7 days, and 30 days (*p* = 1.00, 0.513, 0.910) - hs-CRP at 24 h lower (3.13) vs. controls (4.85) (*p* = 0.048)	No
Normobaric oxygen	high-flow normobaric oxygen (FiO2 50%, flow 15 L/min) for 6 h after recanalization	- mRS at 90 days	- mRS 0–2 at 90 days - NIHSS at 24 h - Infarct volume - Mortality - sICH	- Adjusted common odds ratio for improvement of 1 point mRS at 90 days 2.2 (95% CI, 1.26 to 3.87; *p* = 0.006) - Lower mortality vs. controls (adjusted risk ratio, 0.35; 95% CI, 0.13–0.93; *p* = 0.012). - Infarct volume 9.4 vs. 20.5 ml in controls (adjusted beta coefficient, −20.24; 95% CI, −35.93 to −4.55; *p* = 0.035)	No
Transcranial direct current stimulation	1.5 mA current for 20 min epochs, delivered every hour over a period of 6 h and 20 min	- Infarct growth at 24 h (MRI DWI)	- NIHSS at 7 days - mRS 0–2 at 90 days	- Infarct growth 5.4 vs. 8.3 ml in controls (*p* = 0.28) - NIHSS at 7 days −4.4 vs. −6.2 in controls (*p* = 0.39) - mRS 0–2 63.6 vs. 43.5% in controls (*p* = 0.18)	No

*GOS, Glasgow outcome scale; ITT, Intention to treat; BI, Barthel index; NIHSS, National institutes of health stroke scale mRS, modified Rankin scale; SSS, Scandinavian stroke scale; SAE, serious adverse event; OR, odds ratio; CI, confidence interval; CMH, Cochran–Mantel–Haenszel; QOL, quality of life; MMP, matrix metalloproteinase; TICI, thrombolysis in cerebral infarction*.

**Table 3:** Cochrane risk of bias assessment.

**Study**	**Agent**	**Random sequence generation**	**Allocation concealment**	**Selective reporting**	**Other sources of bias**	**Blinding (outcome)**	**Blinding (participants and personnel)**	**Incomplete outcome data**
Ogawa et al. (12)	Ebselen	Low	Unclear	Unclear	Low	Low	Unclear	Low
Clark et al. (13)	Nalmefene	Unclear	Low	High	High	Unclear	Low	Unclear
Sacco et al. (14)	Gavestinel	Low	Low	Unclear	Low	Low	Low	Unclear
Lyden et al. (15)	Clomethiazole	Low	Low	Unclear	High	Unclear	Low	Low
Krams et al. (16)	UK 279,276	Low	Low	Unclear	Unclear	Low	Low	Low
Amaro et al. (17)	Uric acid	Low	Unclear	Unclear	Unclear	Low	Unclear	Unclear
Diener et al. (18)	NXY-059	Low	Unclear	Unclear	Unclear	Unclear	Low	Low
Ehrenreich et al. (19)	Erythropoietin	Low	Low	Unclear	Low	Low	Low	Low
Teal et al. (20)	Repinotan	Unclear	Unclear	Unclear	Low	Unclear	Low	Unclear
Hemmen et al. (21)	Hypothermia	Low	High	Low	Low	Unclear	High	Unclear
Heiss et al. (22)	Cerebrolysin	Unclear	Unclear	Unclear	High	Low	Unclear	High
Ginsberg et al. (23)	Albumin	Low	Low	Unclear	Low	Low	Low	High
Lang et al. (24)	Cerebrolysin	Low	Unclear	Unclear	Unclear	Unclear	Low	High
Hougaard et al. (25)	Remote ischemic perconditioning	Low	Unclear	Unclear	Unclear	Low	High	High
Saver et al. (26)	Magnesium	Low	Unclear	Unclear	Low	Low	Low	Low
Woodhouse et al. (27)	Transdermal Glyceryl Trinitrate	Low	Low	High	Low	Low	Low	Low
Montaner et al. (28)	Simvastatin	Low	Low	Unclear	Unclear	Low	Low	Low
Wahlgren et al. (29)	Imatinib	Low	Unclear	Unclear	Unclear	Unclear	Unclear	Low
Wang et al. (30)	Epigallocatechin gallate	Low	Unclear	Unclear	Low	Low	Low	Unclear
Chamorro et al. (31)	Uric acid	Low	Low	Unclear	Low	Low	Low	Low
Lyden et al. (32)	3K3A-APC	Low	Unclear	Low	Unclear	Low	Low	Low
Culp et al. (33)	Dodecafluoropentane	Unclear	Low	Unclear	Unclear	Low	Low	Unclear
Kim et al. (34)	Otaplimastat	Low	Low	Unclear	Low	Unclear	Low	Low
Pico et al. (35)	Remote ischemic preconditioning	Low	Low	Unclear	Low	Unclear	Low	Low
Hill et al. (36)	Nerinetide	Low	Low	Unclear	Low	Low	Low	Low
An et al. (37)	Remote ischemic perconditioning	Low	High	Unclear	Unclear	Unclear	High	Unclear
Modrau et al. (38)	Theophylline	Low	Unclear	Unclear	High	Unclear	Unclear	Unclear
He et al. (39)	Remote ischemic perconditioning	Low	Unclear	Unclear	Unclear	Low	Unclear	Low
Cheng et al. (40)	Normobaric oxygen	Low	Low	Unclear	Unclear	Low	Low	Low
Pruvost-Robieux et al. (41)	Transcranial stimulation	Low	Low	Low	Low	Unclear	Low	Low

## Studies Combining Neuroprotection and IVT

### Excitotoxicity

#### Clomethiazole

Clomethiazole is a positive allosteric modulator of GABA (42). The CLASS-T trial (15) tested the effect of clomethiazole in patients concomitantly treated with IVT. All patients (*n* = 190) received IVT within 3 h of stroke onset, and 53% of patients had total anterior circulation stroke (TACS). No effect of clomethiazole was found on the main clinical outcome: the Barthel Index score. In the TACS subgroup, 53% of the clomethiazole patients scored a BI >60 vs. 45% of controls (OR 1.4, 95%CI 0.6–3.2). MRS score distributions were not reported.

#### Magnesium

The use of pre-hospital intravenous magnesium was investigated in 2015 (26). In total 1700 patients were treated, of whom 73% (*n* = 1,246) had ischemic stroke. Of these patients, 36% received IVT. Main outcome was the mRS at 90 days. Mean mRS scores did not differ between the magnesium group and controls (2.7 in each group). Good functional outcome was observed in 52.4% of the magnesium treated group and 52.8% in controls (OR 1.0; 95%CI 0.8–1.2).

#### Transdermal Glyceryl Trinitrate

Transdermal glyceryl trinitrate (GTN), a nitric oxide donor, was tested in patients presenting with symptoms of stroke in 2015 (43). Additional analyses were published on a predefined subgroup of early treated patients (<6 h) in this trial (27). All types of stroke were included, of which 76% (*n* = 208) was ischemic stroke. One third of AIS patients was treated with IVT. Patients that randomized early to GTN had a significant shift toward a lower mRS (adjusted common OR 0.5; 95%CI 0.3–0.8). Good functional outcome was 48,6% in the GTN group and 39,5% in controls (OR 1.4; 95%CI 0.9–2.3). No subgroup analysis for patients treated with IVT was performed.

#### Repinotan

Repinotan is a serotonin (5-HT)1A receptor agonist. Its intravenous use was investigated in 681 stroke patients (20). In addition, 61% of patients was treated with IVT (stratified during inclusion). Primary outcome was the BI, secondary outcomes included the mRS. The proportion of patients with a BI>85 at 3 months was 37 vs. 42% in controls (OR 0.8, 95%CI 0.6–1.1) and 32.4% of patients had an mRS of 0–2 at 3 months vs. 37.8% in controls (OR 0.8, 95%CI 0.6–1.1).

### Oxidative Stress

#### NXY-059

NXY-059 is a free radical trapping agent. Two large, RCTs (18) included a total of 4946 patients who were treated with intravenous NXY-059 within 6 hof stroke onset; over one third received IVT as well. Primary outcome was the mRS at 3 months. Among patients treated with IVT, there was no association between NXY-059 treatment and mRS score (mRS 0–2 43.0 vs. 42.4%, OR 1.1; 95%CI 0.9–1.1).

#### Cerebrolysin

Cerebrolysin is a porcine brain-derived preparation of low-molecular-weight neuropeptides with free amino acids that show pharmacodynamic properties similar to those of naturally occurring neurotrophic factors. In 2013, cerebrolysin was studied in 119 patients, all treated with concomitant IVT (24). Primary outcome was the mRS at 90 days. The study was halted prematurely because of futility after the third interim analysis, showing no significant differences between cerebrolysin and placebo (mRS 0–2 67.2 vs. 66.1%, OR 1.1; 95%CI 0.5–2.3).

#### Simvastatin

In 2016 simvastatin was investigated treating 104 patients with AIS, randomized between orally administered simvastatin or placebo for 90 days (28). In total, 53% of patients were treated with IVT. Primary outcome was the mRS. The trial yielded neutral results (mRS 0–2 at 3 months: adjusted OR 1.0, 95%CI 0.4–2.8). A *post-hoc* analysis showed a possible benefit for patients treated with IVT, with a higher proportion of patients improving >7 points on the NIHSS (adjusted OR 4.1, 95%CI 1.2–14.4).

#### Epigallocatechin Gallate

Epigallocatechin gallate (EGCG) is a naturally occurring polyphenol from green tea extracts. It was tested in 371 AIS patients all treated with IVT (30). The trial reported positive results with lower NIHSS scores in the EGCG treated group in patients treated 3–5 h compared to placebo, though it is unclear whether these differences were significant since no ratios or confidence intervals were reported. MRS scores were not reported.

#### Ebselen

Ebselen is a lipid-soluble seleno-organic compound that was tested in 99 stroke patients for its possible neuroprotective properties reducing oxidative stress (12). It was administered orally within 12 h of stroke onset. More than two thirds of patients were treated with IVT. Primary outcome was CT infarct volume and the Glasgow Outcome Scale. Mean CT infarct volume was 11.4 ml in Ebselen treated patients vs. 13.4 in controls (*p* = 0.099). No mRS scores were reported.

### Inflammation

#### Erythropoietin

The use of recombinant human erythropoietin (EPO) was studied in 2009 (19). Within 6 h of AIS symptom onset, EPO was infused intravenously in 522 patients. Almost two thirds of patients were treated with IVT, which was stratified for in the inclusions. Subgroup analysis revealed that the ΔNIHSS (Day 1 minus Day 90) showed a larger improvement with EPO than with placebo in the non-IVT-subgroup (mean difference of 5.3 ± 5.3 in EPO vs. 3.2 ± 6.4 in placebo; *P* = 0.03). This difference was not observed in the IVT subgroup. The trial raised safety concerns as there were significantly more deaths in the EPO treated group than in controls (16.4 vs. 9.0%, OR 2.0; 95%CI 1.2–3.4). Overall, the trial did not show a significant effect on any of the efficacy outcomes including mRS (mRS 0–2 39.9 vs. 41.1%, OR 1.1; 95%CI 0.8–1.7).

### Cellular Apoptosis

No studies fulfilled the inclusion criteria.

### Other/Non-pharmacological Mechanisms

#### Hypothermia

ICTuS-L tested induced hypothermia at 33°C in AIS patients who also received IVT (21). In total 58 patients were enrolled and 48 (79%) were treated with IVT. Though the primary objective of the study was to investigate safety, assessed efficacy outcomes were NIHSS and mRS. There were no differences in outcome, neither in the overall population, nor in subgroups according to IVT. At 3 months, 18% of patients in the hypothermia group had a mRS score of 0–1 vs. 24% in controls (*P* < 0.77). No OR for good clinical outcome was calculated as exact data on the distribution of mRS scores was not reported.

#### Remote Ischemic Perconditioning

Remote ischemic perconditioning (rPerc) is inducing ischemia in one organ to increase tolerability in another. Four trials investigated the use of rPerc in stroke patients receiving reperfusion therapy: two combining rPerc with IVT, and two trials also treating patients with EVT (see paragraph on neuroprotection and EVT). In 2020 a single center trial (*n* = 68) reported significant results in a trial of rPerc in which patients with AIS were treated with IVT and subsequent ischemic perconditioning (37). Excellent recovery (mRS 0–1) at 3 months was obtained in 72% of patients in the rPerc group vs. 50% in controls (adjusted risk ratio, 9.85; 95%CI, 1.5 to 63.2), though patients with severe stroke (NIHSS >24) were excluded from this trial. A second trial investigated rPerc in 49 patients all treated with IVT (39). Primary outcome was the occurrence of hemorrhagic transformation or other adverse events. Secondary outcome measures included the ratio of mRS 0–1 at 90 days, distribution of mRS, and NIHSS scores. There were no differences in recovery between groups treated with and without rPerc (mRS 0–1 62.5 vs. 68.0% in controls, *p* = 0.69).

## Studies Combining Neuroprotection and EVT (With or Without IVT)

### Excitotoxicity

#### Nerinetide

The eicosapeptide nerinetide was investigated in the NA-1 trial, comparing nerinetide treatment with placebo in 1105 patients with LVO eligible for EVT (36). The primary outcome was favorable outcome defined as an mRS 0–2 at 90 days. Concomitant alteplase was administered in 60% of patients. The primary outcome was achieved by 337 (61%) of 549 patients in the nerinetide group and 329 (59%) of 556 in controls (adjusted RR 1.0, 95%CI 1.0–1.1). Successful recanalization (eTICI ≥2b) was achieved in 86% of the placebo group and 87% of the nerinetide group. The average time between administration of nerinetide and reperfusion was 22 min. In a predefined *post-hoc* subgroup analysis, nerinetide was significantly associated with favorable outcome (risk ratio 1.2 (1.0–1.4) in the non-IVT subgroup (*n* = 446) and not in the IVT subgroup (*n* = 659). The authors hypothesize that an interaction between nerinetide and the use of IVT might have nullified treatment effect with nerinetide in the IVT subgroup.

### Oxidative Stress

#### Albumin

Intravenous administration of albumin in 422 AIS patients was reported in 2013 (23). The majority of patients received IVT (68%), with a small subgroup receiving additional EVT (16%) or EVT alone (4%). Primary endpoint was favorable outcome (mRS 0–1, NIHSS 0–1, or both). The trial was halted prematurely because of futility after an unscheduled third interim analysis. The primary outcome was adjusted for the use of IVT and baseline NIHSS and did not differ between patients in the albumin group and controls (44.0 vs. 44.0%; risk ratio 1.0; 95%CI 0.8–1.1). The proportion of patients with good functional outcome did not differ between treatment groups (56.7 vs. 57.0%, OR 1.0; 95%CI 0.7–1.3). No subgroup analysis was done for the group of patients treated with EVT, and rates of successful recanalization were not reported.

#### Uric Acid

Uric acid is a naturally occurring antioxidant. A pilot study was conducted in 2007 treating 16 patients with uric acid after IVT proving its safety (17). The URICO-ICTUS trial consequently studied a population of 411 stroke patients (44). In 2017 results from a prespecified subgroup analysis were reported with 45 patients who received IVT and EVT and were all treated with intravenous uric acid or placebo within 4.5 h of stroke onset (31). Recanalization was achieved in >80% of patients. Primary outcome was good functional outcome (mRS 0–2) and was observed in 16/24 (67%) patients treated with uric acid and 10/21 (48%) in controls (adjusted OR, 6.1, 95%CI 1.1–34.6). The unadjusted OR analysis did not show a significant effect (OR 2.0 95%CI 0.7–7.3) of uric acid on good functional outcome.

#### Dodecafluoropentane

Dodecafluoropentane (DDFPe) is an oxygen transporting nanodroplet with neuroprotective properties (33). In 2019 administration in 24 patients with AIS was investigated, starting infusion within 12 h of stroke onset (44). Three different dose tiers were used. In total of 16/18 DDFPe treated patients received reperfusion therapy (IVT = 61%, EVT 6%, IVT + EVT 22%). Outcomes were (serious) adverse events, NIHSS and mRS scores. When only the high-dose group of DDFPe was compared to placebo, there was an improvement in both 30- and 90-day mRS (*P* = 0.01 and *P* = 0.03, respectively). No OR for good clinical outcome was calculated as exact data on the distribution of mRS scores were not reported. Sample sizes were too small for subgroup analyses in patients with LVO.

#### Theophylline

Theophylline is speculated to be neuroprotective due to its cerebral vasoactive properties (31). It was investigated as add-on to IVT in 64 patients with AIS (38). All patients received theophylline intravenously within 30 min of IVT and 14% of patients (*n* = 9) was also treated with EVT. A limited number of patients with LVO was included as the majority of patients with severe stroke was unable to provide informed consent. Outcomes were NIHSS improvement and infarct volume growth at 24 h. After 3.5 years the steering committee decided to stop the trial due to slow recruitment. Patients treated with theophylline improved on the NIHSS by 4.7 points (SD, 5.6) compared with an improvement of 1.3 points (SD, 7.5) after IVT alone (*P* = 0.044), though results did not reach significance after correction for multiplicity [adjusted estimated improvement 3.6 (95%CI, 0.1–7.1)]. The corresponding adjusted treatment effect on infarct growth was 35.2% (95%CI, −17.4–87.7). Good functional outcome did not differ between treatment groups (81% in both groups, OR 1.1; 95%CI 0.3–3.8). No treatment effect was found in the subgroup treated with EVT.

### Inflammation

#### Otaplimastat

Otaplimastat is a cerebroprotectant that inhibits the matrix metalloprotease pathway (34). It was tested in AIS patients receiving IVT in 2019 (34). The trial was divided in a safety trial (*n* = 11) and randomized controlled efficacy trial (*n* = 69). The latter part compared two dose tiers (40/80 mg) vs. placebo. In total 29% of patients underwent EVT in addition to IVT. Primary endpoints were intracranial hemorrhage, systemic bleeding and the mRS. No significant differences in the occurrence of hemorrhagic transformation were found between groups. A non-significant shift in distribution of mRS was reported in the otaplimastat 40 mg group vs. placebo with an adjusted OR of 3.2 (95%CI 0.9–10.9). Good functional outcome did not differ between groups (75.0 vs. 76.2%, OR 0.9; 95%CI 0.3–3.2). Of patients treated with EVT, 77% achieved good recanalization (mTICI score ≥2b).

#### 3K3A-APC

3K3A-APC is a modified version of Activated Protein C. The RHAPSODY trial investigated its intravenous use in treating AIS in combination with IVT (54%), EVT (5%) or both (42%) in 110 patients (32). Primary outcome was safety (maximum tolerated dose), secondary outcomes included mRS and NIHSS. There were no patients with an mRS ≥2 in both the 3K3A-APC group and control group (mRS 0: 88 vs. 93%, mRS 1: 12 vs. 7%).

### Cellular Apoptosis

#### Imatinib

Imatinib is a tyrosine kinase inhibitor which reduces cellular apoptosis by blocking the signaling of platelet-derived growth factor alpha. It was investigated in 60 stroke patients in three different dose levels (400, 600, and 800 mg, orally) (29). All patients received IVT and 42% of patients underwent EVT. Primary outcome measure was the occurrence of adverse events. Secondary outcomes included infarct volume, NIHSS improvement and good functional outcome (mRS 0–2). Treating AIS patients with imatinib was safe. Patients treated with imatinib had more NIHSS improvement than controls with a significant difference measured in the high dose group, adjusted for treatment with EVT [low, medium, and high dose: 2 points (*p* = 0.259), 3 points (*p* = 0.106), and 5 points (*p* = 0.012) respectively]. The mean NIHSS improvement adjusted for treatment with EVT was 0.6 per 100 mg imatinib (−0.6; 95%CI −1.1 to −0.1). Good functional outcome did not differ between groups (69.2% in all treatment groups vs. 61.0% controls, OR 1.2; 95%CI 0.4–4.1).

### Other/Non-pharmacological Mechanisms

#### Remote Ischemic Perconditioning

Two trials investigated the use of rPerc in stroke patients treated with EVT. The first trial investigated the pre-hospital use of rPerc in 443 stroke patients (25). All MRI proven stroke patients who underwent IVT were analyzed. Primary end point was penumbral salvage (volume of perfusion–diffusion mismatch not progressing to infarction after 1 month). No treatment effect of rPerc was found in median penumbral salvage (IQR): 11.9 mL (0.5–63.4) vs. 14.1 mL (1.6–79.8) nor good functional outcome (80 vs. 88%, OR 0.5; 95%CI 0.3–1.2). Though the authors report some patients being treated by EVT, no exact data about this is presented and no subgroup analyses were done. The second trial investigated in-hospital rPerc in 188 patients of who 87% received IVT and 34% received EVT (35). No differences were reported in terms of infarct growth (MRI) or functional outcome (NIHSS 24 h, BI 90 days). An excellent outcome (mRS 0–1) was observed in 46 patients (51%) in the intervention group vs. 37 (41%) in controls (OR 1.6; 95%CI 0.9–2.9).

#### Normobaric Oxygen

Normobaric oxygen (NBO) potentially reduces secondary injury to penumbral tissue by reducing oxidative stress. The use of high-flow NBO (FiO2 50%, 15 L/min) was investigated in 175 stroke patients all treated with EVT (40). NBO was administered for 6 h following the EVT procedure. In addition, 45% of patients were treated with IVT. Stroke patients without recanalization (TICI score 0) after EVT were excluded. Primary outcome was the mRS, secondary outcomes included NIHSS at 24 h, infarct volume, and mortality. The common OR of NBO treatment indicating the improvement of 1 point on the mRS was 2.2 (95%CI, 1.3–3.9). In NBO treated patients, mortality was lower (adjusted risk ratio, 0.4; 95%CI 0.1–0.9) and NBO treated patients had smaller final infarct volumes (9.4 vs. 20.5 ml in controls (adjusted beta coefficient, −20.2; 95%CI −35.9 to −4.6). The proportion of patients with good functional outcome was 64.8% in the NBO treated group and 50.6% in controls (OR 1.4; 95%CI 0.7–1.6).

#### Transcranial Stimulation

Cathodal transcranial direct current stimulation (C-tDCS) is a non-invasive neurostimulation method modulating the excitability of cortical neurons (41). It was investigated in acute stroke patients in 2021 (41). In total 45 patients were treated with C-tDCS or sham procedure, in 6 sessions of 20 min. Most patients (42/45) were treated with IVT, and over one third (20/45) (also) with EVT. The primary outcome was growth of infarct volume at 24 h. Secondary outcomes were NIHSS score at 7 days and mRS 0–2 at 90 days. No benefit of C-tDCS was found on any of the primary or secondary outcomes (mRS 0–2 63.6 vs. 43.5% in controls, OR 2.3; 95%CI 0.7–2.5).

## Discussion

This systematic review investigates effects of neuroprotective agents in combination with reperfusion therapy in AIS. Our search yielded 30 studies in which neuroprotective agents were tested. Only 15 studies specifically included patients treated with IVT and/or EVT and were designed to investigate effects of neuroprotective treatment in addition to reperfusion therapy. Of these 15 studies, five reported positive results of treatment on various clinical outcome measures as chosen by the investigators. These results indicate a high potential for some neuroprotective treatments in addition to reperfusion. Studies investigating neuroprotective treatment in addition to IVT that report a positive effect of neuroprotection on clinical outcome are the trials investigating GTN, imatinib and remote ischemic perconditioning (rPerc). Unfortunately, two thirds of patients did not receive reperfusion therapy in the GTN trial. Early treatment of stroke patients with GTN was recently investigated in the prehospital transdermal glyceryl trinitrate in patients with ultra-acute presumed stroke (RIGHT-2) trial (45). The trial did not show a beneficial effect of GTN in pre-hospital treatment of stroke. Since no subgroup analyses on patients treated with IVT were reported this study fell outside the scope of our current search. The trial investigating imatinib was primarily a safety trial with a limited number of patients. A follow up trial is currently ongoing and estimated to be completed in 2023. One of the four trials on rPerc found a larger probability of excellent outcome (mRS 0–1) with rPerc than without rPerc. These results should be interpreted with caution since outcome assessment in this trial was not blinded and the study was susceptible to allocation concealment bias.

Studies investigating neuroprotective treatment in addition to EVT that report a significant effect of neuroprotection on clinical outcome are the trials in which patients were treated with 1) the combined treatment of IVT and EVT with uric acid, 2) the use of nerinetide in stroke patients treated with EVT, and 3) the use of high-flow NBO in stroke patients successfully treated with EVT. The article on uric acid reported results from a predefined subgroup analysis with patients treated with IVT and EVT. All three studies were methodologically sound with a low risk of bias. A new trial investigating nerinetide (ESCPAPE-NEXT) is already recruiting patients to confirm these results (NCT04462536). A follow-up trial for uric acid has been planned as well (11).

By limiting our search to RCTs with the terms *neuroprotection* (and variants) and *stroke*, it is possible that some trials on neuroprotective agents and reperfusion therapy in AIS were not found. Indeed, by cross-checking references, two more articles were added to our results. A large number of neuroprotective agents has been investigated in AIS in the past. It is possible that a number of articles fell outside our search, despite the use of reperfusion therapy, simply because no data on the use of reperfusion therapy were reported. As we limited our search strategy to neuroprotection trials using *efficacy* as outcome, safety trials may have fallen outside the scope of our search. Different studies reported various outcome measures. Our calculated ORs for good clinical outcome (mRS 0–2) did not reach statistical significance in any of the reported trials. The heterogeneity in outcome, as well as variations in treatment administration (e.g. timing, dosage), and the lack of replication-studies impaired the development of a quantitative synthesis. Future studies should aim to report standardized functional outcome measures, most notably the mRS, as was proposed by the Stroke Therapy Academic Industry Roundtable (STAIR) (46), as well as strive to replicate and validate promising findings.

Recanalization rates in stroke patients treated with EVT are higher (60–90%) (4) than in patients treated with IVT (10–30%) (47, 48). Neuroprotective treatment may be more effective in these patients. As EVT is a relatively new treatment, a very limited number of neuroprotective agents has been tested in this setting. Future research should seek to combine EVT with neuroprotective strategies to test the hypothesis that neuroprotective treatment in combination with successful reperfusion leads to better outcomes in patients with AIS. Rapid reperfusion can, however, also lead to reperfusion injury, induced by high levels of free oxygen radicals, when oxygen becomes again available in previous hypoxic-ischemic tissue. For that reason treatments, which target reduction in oxidative stress might be beneficial in the prevention of this reperfusion injury.

An overview of ongoing studies investigating neuroprotective agents in addition to EVT using efficacy outcome measures is shown in Table 4. Future pre-clinical research is needed to improve the targeting of neuroprotective agents as well as their action mechanism. STAIR X proposed the term neurovascular unit (NVU) to better recognize different parts of the brain susceptible to ischemia. The NVU consists of various cell types including astrocytes, neurons and endothelial cells with a differential vulnerability to ischemic injury. New research strategies may focus on neuroprotective strategies targeting one or multiple parts of the NVU (13). Indeed, the importance of solid pre-clinical research in neuroprotective strategies has already been recognized by the initiation of the Stroke Preclinical Assessment Network (SPAN), a project started by the National Institutes of Health (NIH) in 2018 in which 7 different laboratories seek to employ rigorous, clinical research practices while assessing the effectiveness of potential neuroprotective therapies in reducing ischemic brain injury. New pre-clinical research strategies may focus on neuroprotective strategies targeting one or multiple parts of the NVU.

**Table 4:** Current trials combining neuroprotection and reperfusion.

**Study**	**Acronym**	**Agent**	**Primary outcome**	**Secondary outcome**	**Reperfusion technique**	**Estimated completion**	**NCT number**
A Randomized Controlled Trial Assessing the Efficacy and Safety of Normobaric Hyperoxia for Acute Ischemic Stroke Patients Undergoing Endovascular Treatment	OPENS-2	Oxygen	mRS	Cerebral infarct volume, proportion of mRS 0–1 at 3 months, proportion of mRS 0–3 at 3 months, NIHSS, BI, NIHSS improvement, EuroQol- 5D, MOCA, mortality, ICH	EVT +− IVT	2022	NCT04681651
Transcranial Electrical Stimulation in Stroke EaRly After Onset Clinical Trial_ Bridging and Adjunctive Neuroprotection	TESSERACT-BA	Transcranial stimulation	Safety	mRS at 90 days , infarct volume, 3-month EuroQol- 5D	EVT	2022	NCT04061577
Efficacy of Cerebrolysin Treatment as an add-on Therapy to Mechanical Thrombectomy in Acute Ischemic Stroke.	N/A	Cerebrolysin	mRS	Distribution of mRS, proportion of NIHSS at day 7, 30, 90, mortality, infarct volume, ICH	EVT +− IVT	2023	NCT04904341
Regional Hypothermia in Combination With Endovascular Thrombectomy in Acute Ischemic Stroke	RE-HIBER	Hypothermia	Safety	ICH, mortality, NIHSS at 24 h, 7 days, mRS at 90 days	EVT +− IVT	2021	NCT04554797
Safety and Optimal Neuroprotection of neu2000 in Ischemic Stroke With Endovascular reCanalization	SONIC	Neu2000	mRS	Distribution of mRS, ratio of NIHSS 0–2, BI, hemorrhagic transformation within last study treatment day (4 or 5)	EVT +− IVT	unknown	NCT02831088
Feasibility and Safety Study to Evaluate the Neuroprotective Effect of Hemodialysis in Acute Ischemic Stroke	DIAGLUICTUS2	Hemodialysis	Safety, feasibility	Reduction of plasma pro-inflammatory cytokines and reduction of plasma Glu after 1st and 2nd session of hemodialysis, evolution of NIHSS day 7, mRS at 90 days, infarct volume	EVT +− IVT	2022	NCT04297345
Neuroprotective Effect of Remote Ischemic Conditioning in Ischemic Stroke Treated With Mechanical Thrombectomy	PROTECT I	Ischemic preconditioning	Infarct volume	NIHSS 7 days, mRS 90 days, infarct core change (MRI), TICI	EVT +− IVT	2022	NCT03915782
Intra-arterial Neuroprotective Strategy for Ischemic STroke Patients With Endovascular Therapy	INSIST-TE	Butylphthalide	Safety	mRS at 90 days, proportion of mRS 0–1, proportion of mRS 0–2, NIHSS decrease at 48 h, ICH	EVT +− IVT	2021	NCT04664933
Improving Neuroprotective Strategy for Ischemic Stroke After Thrombectomy Followed by DELP	INSIST-DELP	Delipid Extracorporeal Lipoprotein filter from Plasma	Proportion of mRS 0–2 at 90 days	Proportion of mRS 0–1 at 90 days, NIHSS decrease 48 h/7 days, infarct volume, ICH	EVT +− IVT	2022	NCT04708730
Improving Neuroprotective Strategy for Ischemic Stroke With Sufficient Recanalization After Thrombectomy by Intra-arterial Cocktail Therapy	INSIST-CT	Argatroban, edaravone, and glucocorticoid	Proportion of mRS 0–2 at 90 days	Proportion of mRS 0–1, early neurological improvement (NIHSS −4 at 48 h)	EVT +− IVT	2021	NCT04202549
Improving Neuroprotective Strategy for Ischemic Stroke With Sufficient Recanalization After Thrombectomy by Edaravone Dexborneol	INSIST-ED	Edaravone dexborneol	Proportion of mRS 0–2 at 90 days	Proportion of mRS 0–1 at 90 days, distribution of mRS at 90 days, NIHSS change at 24 h/48 h/2 wk, infarct volume	EVT +− IVT	2021	NCT04667637
Edaravone Dexborneol for Treatment of Acute Ischemic Stroke With Endovascular Therapy in Extended Time Windows	EXISTENT	Edaravone dexborneol	Proportion of mRS 0–3 at 90 days, mTICI, sICH	Change in NIHSS at 48 h/7 d/90 d, distribution of mRS, change infarct volume, BI>95 at day 14/30/90	EVT	2022	NCT04817527
Efficacy and Safety of Butylphthalide for Acute Ischemic Stroke Patients Receiving Intravenous Thrombolysis or Endovascular Treatment	BAST	Butylphthalide	Favorable outcome (By NIHSS category, mRS)	NIHSS change at 2 wk/90 d, infarct volume, recanalization rate	EVT +− IVT	2022	NCT03539445
Remote Ischemic Conditioning Paired With Endovascular Treatment for Acute Ischemic Stroke	REVISE-2	Remote ischemic perconditioning	Infarct volume	mRS at 90 days, NIHSS change at 90 days, ICH	EVT +− IVT	2023	NCT03045055
Imatinib in Acute Ischaemic Stroke	N/A	Imatinib	Proportion of mRS 0–2 at 90 days	Change in mRS score at 3 months compared to baseline at 90 days, ICH, grade of ICH, mortality	EVT +− IVT	2023	NCT03639922

*source: Clinicaltrials.gov mRS, modified Rankin Scale; NIHSS, National Institutes of Health Stroke Scale (s); ICH, (symptomatic) intracranial hemorrhage; mTICI, modified Thrombolysis in cerebral infarction; BI, Barthel Index; MOCA, Montreal Cognitive Assessment*.

## Conclusion

Studies specifically investigating neuroprotection in addition to reperfusion treatment are scarce. Five of 15 studies report a positive treatment effect of neuroprotection in addition to reperfusion therapy. Positive results are reported for combining EVT and IVT with uric acid, nerinetide in addition to EVT without IVT, rPerc with IVT, and the use of imatinib and normobaric oxygen in patients treated with EVT and/or IVT, although various clinical outcome measures were reported and validation of these results needs to be established. Future neuroprotection studies should combine neuroprotective agents with reperfusion therapies in AIS or include prespecified subgroup analyses for treatment with IVT and/or EVT.

## Data Availability Statement

The original contributions presented in the study are included in the article/Supplementary Material, further inquiries can be directed to the corresponding author.

## Author Contributions

EV, VG, and IW contributed to conception and design of the review. EV wrote the first draft of the manuscript. EV and VG performed the statistical analyses. VG, AL, DD, MW, JH, AE, YR, CP-S, and IW wrote sections of the manuscript. All authors contributed to manuscript revision, read, and approved the submitted version.

## Funding

This open access publication fee was funded by a grant from the Research Fund of Haaglanden Medisch Centrum.

## Conflict of Interest

CP-S is founder and consultant at Neurophyxia BV. She holds several patents and stocks of Neurophyxia BV. DD received grants from Dutch Heart Foundation, Brain Foundation Netherlands, The Netherlands Organisation for Health Research and Development, Health Holland Top Sector Life Sciences & Health, Stryker, Penumbra, Inc, Medtronic, Thrombolytic Science LLC, and Ceronovus. YR is a shareholder at Nicolab B.V. AL received grants from Dutch Heart Foundation, Brain Foundation Netherlands, The Netherlands Organisation for Health Research and Development, Health Holland Top Sector Life Sciences & Health, Stryker, Penumbra, Inc, Medtronic, Thrombolytic Science LLC, and Ceronovus. MJHW received grants from Dutch Heart Foundation, Brain Foundation Netherlands, The Netherlands Organisation for Health Research and Development and an unrestricted research grant from Electrocore. The remaining authors declare that the research was conducted in the absence of any commercial or financial relationships that could be construed as a potential conflict of interest.

## Publisher's Note

All claims expressed in this article are solely those of the authors and do not necessarily represent those of their affiliated organizations, or those of the publisher, the editors and the reviewers. Any product that may be evaluated in this article, or claim that may be made by its manufacturer, is not guaranteed or endorsed by the publisher.
