# Supplementary material for: Systematic Review - Combining Neuroprotection With Reperfusion in Acute Ischemic Stroke
Source: Front Neurol. 2022 Mar 17;13:840892. doi: 10.3389/fneur.2022.840892 (PMC8969766; doi:10.3389/fneur.2022.840892)
Supplement: Supplementary file 1 [file Data_Sheet_1.DOCX]

**SM1: search query**

| Box 1: Search query  (("ischemic stroke"[tw] OR "ischemic strokes"[tw] OR "ischaemic stroke"[tw] OR "ischaemic strokes"[tw] OR (("Brain Ischemia"[Mesh] OR "Brain Ischemia"[tw] OR "Brain Ischaemia"[tw] OR "Cerebral Ischemia"[tw] OR "Cerebral Ischaemia"[tw] OR "Brain Hypoxia-Ischaemia"[tw] OR "Brain Hypoxia-Ischemia"[tw] OR "Brain Infarction"[tw] OR "Brain Infarctions"[tw] OR "Brain Stem Infarction"[tw] OR "Brain Stem Infarctions"[tw] OR "Cerebral Infarction"[tw] OR "Cerebral Infarctions"[tw] OR "Subclavian Steal Syndrome"[tw] OR "Transient Ischemic Attack"[tw] OR "Transient Ischemic Attacks"[tw] OR "Transient Ischaemic Attack"[tw] OR "Transient Ischaemic Attacks"[tw] OR "Vertebrobasilar Insufficiency"[tw]) AND ("Stroke"[Mesh] OR "Stroke"[tw] OR "Strokes"[tw] OR "Brain Vascular Accident"[tw] OR "Brain Vascular Accidents"[tw] OR "Cerebrovascular Accident"[tw] OR "Cerebrovascular Accidents"[tw] OR "Cerebrovascular Apoplexy"[tw] OR "CVA"[tw] OR "CVAs"[tw])) OR (("ischemia"[tw] OR "ischaemia"[tw] OR "ischemic"[tw] OR "ischaemic"[tw]) AND ("Stroke"[Mesh] OR "Stroke"[tw] OR "Strokes"[tw] OR "Brain Vascular Accident"[tw] OR "Brain Vascular Accidents"[tw] OR "Cerebrovascular Accident"[tw] OR "Cerebrovascular Accidents"[tw] OR "Cerebrovascular Apoplexy"[tw] OR "CVA"[tw] OR "CVAs"[tw]))) AND ("Neuroprotection"[Mesh] OR "Neuroprotection"[Mesh] OR "neuroprotect*"[tw] OR "Neuro protection"[tw] OR "neuro protect*"[tw] OR "Neuroprotective Agents"[Mesh] OR "Neuroprotective Agents"[Pharmacological Action] OR "(3-aminopropyl)(n-butyl)phosphinic acid"[tw] OR "1-amino-1,3-dicarboxycyclopentane"[tw] OR "1-aminocyclopropane-1-carboxylic acid"[tw] OR "3-aminobenzamide"[tw] OR "3-n-butylphthalide"[tw] OR "4-carboxy-3-hydroxyphenylglycine"[tw] OR "4-methylcatechol"[tw] OR "5-amino-7-(2-phenylethyl)-2-(2-furyl)pyrazolo(4,3-e)-1,2,4-triazolo(1,5-c)pyrimidine"[tw] OR "5-imino-1,2,4-thiadiazole VP1.14"[tw] OR "6-(1H-imidazol-1-yl)-7-nitro-2,3(1H,4H)-quinoxalinedione"[tw] OR "7-((1-alkylpiperidin-3-yl)methoxy)coumarin"[tw] OR "7-chlorokynurenic acid"[tw] OR "7-nitroindazole"[tw] OR "8-((4-chlorophenyl)thio)cyclic-3',5'-AMP"[tw] OR "ACTH (4-7), Pro-Gly-Pro-"[tw] OR "almitrine, raubasine drug combination"[tw] OR "AM 36"[tw] OR "aptiganel"[tw] OR "astressin"[tw] OR "benzyloxycarbonylvalyl-alanyl-aspartyl fluoromethyl ketone"[tw] OR "cerebrolysin"[tw] OR "Chlormethiazole"[tw] OR "Cilostazol"[tw] OR "Dextrorphan"[tw] OR "Dizocilpine Maleate"[tw] OR "DJ-1 based peptide, ND-13"[tw] OR "DRalpha1-MOG-35-55"[tw] OR "ebselen"[tw] OR "Edaravone"[tw] OR "eliprodil"[tw] OR "emopag"[tw] OR "enadoline"[tw] OR "epigallocatechin gallate"[tw] OR "ethyl phenylacetyl-Pro-Gly"[tw] OR "ethylisopropylamiloride"[tw] OR "fructose-1,6-diphosphate"[tw] OR "gacyclidine"[tw] OR "GYKI 52466"[tw] OR "H290-51"[tw] OR "HM01"[tw] OR "HU 211"[tw] OR "huperzine A"[tw] OR "isospaglumic acid"[tw] OR "linopirdine"[tw] OR "lubeluzole"[tw] OR "Meclofenoxate"[tw] OR "mesedin"[tw] OR "Methylprednisolone"[tw] OR "Methylprednisolone Hemisuccinate"[tw] OR "N-(1-methyl-2-phenylethyl)adenosine"[tw] OR "N-(3-propylcarbamoyloxirane-2-carbonyl)-isoleucyl-proline"[tw] OR "nefiracetam"[tw] OR "neurotrophin 4"[tw] OR "neurotrophin 5"[tw] OR "nitroxyl"[tw] OR "nizofenone"[tw] OR "phenyl-N-tert-butylnitrone"[tw] OR "Piracetam"[tw] OR "propentofylline"[tw] OR "rasagiline"[tw] OR "remacemide"[tw] OR "Riluzole"[tw] OR "rimcazole"[tw] OR "Rivastigmine"[tw] OR "RS 5186"[tw] OR "S-Nitrosoglutathione"[tw] OR "Selegiline"[tw] OR "sipatrigine"[tw] OR "tempol"[tw] OR "tenocyclidine"[tw] OR "tirilazad"[tw] OR "tricyclic pyrones"[tw] OR "U 74389F"[tw] OR "U 78517F"[tw] OR "Vasoactive Intestinal Peptide"[tw] OR "vinpocetine"[tw] OR "ziconotide"[tw]) AND ("Drug Therapy"[Mesh] OR "drug therapy"[Subheading] OR "Drug Therapy"[tw] OR "pharmacotherapy"[tw] OR "pharmacotherap*"[tw] OR "pharmacologic therapy"[tw] OR "chemotherapy"[tw] OR "Therapeutic Uses"[Mesh] OR "Pharmaceutical Preparations"[Mesh]) AND ("clinical trial"[pt] OR "controlled trial"[tw] OR "clinical trial"[tw] OR "randomized controlled trial"[pt] OR "controlled clinicl trial"[pt] OR "randomized"[tw] OR "randomised"[tw] OR "placebo"[tw] OR "Placebos"[mesh] OR "clinical trials as topic"[mesh] OR "randomly"[tw] OR "RCT"[tw] OR "trial"[ti]) NOT ("animals"[mesh] NOT "humans"[mesh])) |
| --- |

**SM2: extraction form**


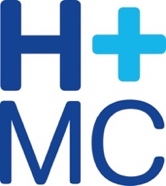


**Extraction form systematic review neuroprotection & reperfusion therapy**

**Article:**

**Author:**

**Journal and year:**

**Agent:**

**Trial name (optional):**

**Center:**

**Date of accession:**

|  |
| --- |

**Excluded?**

Yes  No

**Reason:**

**Number of patients:**

**Intervention:**

**Timing of intervention:**

**Reperfusion strategy:**

IVT  EVT  IVT + EVT

**Number of patients treated with IVT:**

**Number of patients treated with EVT:**

**Number of patients treated with IVT + EVT:**

**Inclusion criteria:**

**Exclusion criteria:**

**Baseline characteristics:**

**Primary outcome:**

**Secondary outcome:**

**Radiological outcome:**

**Results:**

**Large vessel occlusion documented?**

Yes  No

**Recanalization documented?**

Yes  No

**Benefit of neuroprotection?**

Yes  No

**Risk of bias:**

Random sequence generation -  Low  High  Unclear

Allocation concealment **-**  Low  High  Unclear

Selective reporting -  Low  High  Unclear

Other sources of bias -  Low  High  Unclear

Blinding (outcome) -  Low  High  Unclear

Blinding (patients and personnel) -  Low  High  Unclear

Incomplete outcome -  Low  High  Unclear

**SM3: calculated Odds ratios (mRS 0-2 vs 3-6 and mortality)**

|  |  | treatment | control |  | treatment | control |  |
| --- | --- | --- | --- | --- | --- | --- | --- |
|  |  | mRS 0-2 | | OR (95%CI) | mRS 6 (mortality) | | OR (95%CI) |
| Ogawa et al. | Ebselen | - | - | - | 9.3% | 7.1% | 1.3 (0.3-5.7) |
| Clark et al. | Nalmefene | - | - | - | 16% | 15.6% | 1.0 (0.6-1.9) |
| Sacco et al. | Gavestinel | - | - | - | 23% | 19% | 1.2 (0.9-1.6) |
| Lyden et al. | **Clomethiazole** | **-** | - | - | 15% | 10% | 1.7 (0.7-4.1) |
| Krams et al. | UK 279,276 | - | - | - | 17% | 15% | 1.2 (0.8-1.7) |
| Amaro et al. | **Uric acid** | **-** | - | - | 12.5% | 12.5% | 1.0 (0.1-13.0) |
| Diener et al. | NXY-059 | 43% | 42.4% | 1.1 (0.9-1.2) | 16.6% | 16.4% | 1.0 (0.8-1.2) |
| Ehrenreich et al. | Erythropoietin | 39.9% | 41.1% | 1.1 (0.8-1.7) | 16.4% | 9% | 2.0 (1.2-3.4) |
| Teal et al. | Repinotan | 32.4% | 37.8% | 0.8 (0.6-1.1) | 21% | 20% | 1.1 (0.7-1.5) |
| Hemmen et al. | Hypothermia | - | - | - | 21.4% | 16.7% | 1.4 (0.5-5.1) |
| Heiss et al. | Cerebrolysin | - | - | - | 5.3% | 6.6% | 0.9 (0.5-1.5) |
| Ginsberg et al. | Albumin | 56.7% | 57% | 1.0 (0.7-1.3) | 12% | 10% | 1.2 (0.8-1.9) |
| Lang et al. | **Cerebrolysin** | 67.2% | 66.1% | 1.1 (0.5-2.3) | 7.3% | 6.8% | 1.1 (0.3-4.5) |
| Dupont Hougaard et al. | **Remote ischemic perconditioning** | 80% | 88% | 0.5 (0.3-1.2) | 4% | 1% | 2.8 (0.3-25.4) |
| Saver et al. | Magnesium | 52.4% | 52.8% | 1.0 (0.8-1.2) | 15.3% | 15.4% | 1.0 (0.8-1.3) |
| Woodhouse et al. | Transdermal Glyceryl Trinitrate | 48.6% | 39.5% | 1.4 (0.9-2.3) | 7.6% | 20.2% | 0.3 (0.1-0.9) |
| Montaner et al. | Simvastatin | 68.8% | 70% | 0.9 (0.4-2.2) | 6.3% | 6% | 0.7 (0.1-4.3) |
| Wahlgren et al. | **Imatinib** | 69.2% | 61.1% | 1.2 (0.4-4.1) | 0% | 11.1% | 0.1 (0.0-1.8) |
| Wang et al. | **Epigallocatechin gallate** | **-** | - | - | - | - | - |
| Chamorro et al. | **Uric acid** | 66.7% | 47.6% | 2.2 (0.7-7.3) | 8.3% | 4.8% | 1.8 (0.2-21.6) |
| Lyden et al. | 3K3A-APC | 100% | 100% | 1.5 (0.0-76.7) | 13.6% | 13.6% | 1.0 (0.3-3.0) |
| Culp et al. | Dodecafluoropentane | - | - | - | 5.6% | 16.7% | 0.3 (0.0-5.6) |
| Kim et al. | **Otaplimastat** | 75% | 76.2% | 0.9 (0.3-3.2) | 5% | 4.8% | 1.1 (0.1-12.3) |
| Pico et al. | Remote ischemic perconditioning | 68.9% | 65.9% | 1.2 (0.6-2.2) | 15.1% | 10.5% | 1.5 (0.6-3.6) |
| Hill et al. | **Nerinetide** | 61.4% | 59.2% | 1.1 (0.9-1.4) | 12% | 14% | 0.9 (0.6-1.2) |
| Jia-qi An et al. | **Remote ischemic perconditioning** | 81.3% | 58.8% | 3.0 (1.0-9.3)* | 3.1% | 8.8% | 0.3 (0.0-3.4) |
| Modrau et al. | **Theophylline** | 81% | 81% | 1.1 (0.3-3.8) | 0% | 6% | 0.2 (0.0-3.8) |
| Yao-De He et al. | **Remote ischemic perconditioning** | 79.2% | 76% | 1.2 (0.3-4.6) | 4.2% | 0% | 3.3 (0.1-83.9) |
| Zhe Cheng et al. | **Normobaric oxygen** | 64.8% | 50.6% | 1.4 (0.7-2.6) | 5.7% | 19.5% | 0.2 (0.1-0.7) |
| Pruvost-Robieux et al. | Transcranial direct current stimulation | 63.6% | 43.5% | 2.3 (0.7-2.5) | 9.1% | 30.4% | 0.2 (0.0-1.3) |
| Studies with 100% of patients treated with reperfusion therapy * p=0.0522 | | | | | | | |
